# Treatment of Relapsed/Refractory t(11;14) Multiple Myeloma and High‐Risk Myelodysplastic Syndrome With Venetoclax

**DOI:** 10.1155/crh/6491283

**Published:** 2025-12-12

**Authors:** Allison O. Taylor, Stephanie Clune, JoAnn Liu, Cristina Gasparetto, Thomas W. LeBlanc

**Affiliations:** ^1^ Division of Hematologic Malignancies and Cellular Therapy, Department of Medicine, Duke University School of Medicine, Durham, North Carolina, USA, duke.edu; ^2^ Duke Blood Cancer Center, Duke Cancer Institute, Duke University Medical Center, Durham, North Carolina, USA, dukemedicine.org

**Keywords:** high-risk myelodysplastic syndrome, multiple myeloma, Venetoclax

## Abstract

Patients with multiple myeloma (MM) have an inherent risk for secondary myeloid malignancies. Innovative approaches to treatment are needed when these hematologic malignancies co‐occur. Venetoclax (VEN), a BCL2 inhibitor, has been used in combination with Azacitidine in acute myeloid leukemia, in the trial setting in high‐risk myelodysplastic syndrome (MDS), and in t(11; 14) MM in the salvage setting. Here, we present a case report of concurrent treatment of high‐risk MDS and t(11; 14) MM in a patient, using VEN‐based therapy. Following diagnosis with IgG t(11; 14) MM, the patient received treatment with Lenalidomide, Bortezomib, and Dexamethasone and achieved a very good partial response. She subsequently proceeded to autologous stem cell transplant and thereafter continued on Lenalidomide maintenance. Relapsed disease was noted 3 years following transplant, with a concurrent diagnosis of a high‐risk MDS (mutations in NRAS and DNMT3A). Given the known efficacy of VEN in her concomitant malignancies, we elected to use a VEN‐based regimen, at a lower dose of VEN than has been shown to be efficacious in t(11; 14) MM. Bone marrow biopsy demonstrated response from the perspective of both malignancies, until she had relapsed disease with a mixed phenotype acute leukemia ∼ 19 months after relapsed MM/diagnosis of high‐risk MDS. Overall, this case demonstrates successful treatment of both hematologic malignancies at a lower dose of VEN than previously shown to be efficacious in t(11; 14) MM.

## 1. Introduction

Multiple myeloma (MM) is the second most common hematologic malignancy in the United States and is a clonal plasma cell disorder characterized by the abnormal proliferation of plasma cells [1]. Survival in MM has drastically improved in recent decades; however, there is a high risk for secondary malignancies, specifically myeloid malignancies, which negatively impacts overall survival [2]. In a cohort of MM patients, Maliankody et al. noted an 11.5‐fold increased risk for myeloid malignancies (myelodysplastic syndrome/MDS and acute myeloid leukemia/AML) [1]. MM‐associated myeloid malignancies have a worse prognosis, and shorter median overall survival compared to de novo myeloid malignancies; thus, innovative approaches are necessary for treatment [2].

B‐cell leukemia/lymphoma‐2(BCL2) is an antiapoptotic protein necessary for survival of malignant cells. The drug Venetoclax (VEN) inhibits BCL2 and is approved as a low‐intensity regimen in AML and has been studied in high‐risk MDS [3, 4]. In t(11; 14) MM, VEN has been used as monotherapy or in combination with other MM therapies as a salvage therapy, although data on its use in this capacity is mixed [5–9]. Therefore, VEN is a feasible option for the simultaneous treatment of MM and a myeloid malignancy. Here, we describe a case of a patient with t(11; 14) MM who developed high‐risk MDS and received treatment with VEN with dual effectiveness. The patient consented to the submission of this case report.

## 2. Case Description

A 73‐year‐old female with a history of hypertension and coronary artery disease presented with left hip pain. Serum protein electrophoresis with immunofixation demonstrated an IgG lambda M spike of 4.47. Serum free light chains (FLCs) showed a normal FLC kappa at 0.52, increased FLC lambda at 323.89, and a normal FLC kappa/lambda ratio of 0 (Table 1). These findings were concerning for a diagnosis of IgG lambda MM. A bone marrow biopsy demonstrated 80% plasma cells in a 60% cellular marrow, and the accompanying flow cytometry had 11% phenotypically abnormal plasma cells, confirming the diagnosis. Ancillary testing showed abnormal cytogenetics (50, XX with a t(11; 14)), abnormal fluorescent in situ hybridization (FISH) with a CCND1/IGH fusion consistent with t(11;14), a MYC rearrangement (8q24), and three copies of FGFR3 (4p16). She started Lenalidomide, Bortezomib, and Dexamethasone in 11/2018 and achieved a very good partial response (VGPR) after four cycles. Thus, she proceeded to autologous stem cell transplant in 2/2019 and continued on maintenance Lenalidomide.

**Table 1:** Laboratory trends over time pre and post treatment with venetoclax (VEN) and azacitidine (AZA).

Evaluation timing	Hgb (g/dL)	WBC (x10 ^9/L)	Plts (x10^9/L)	BM blasts %	M Spike (g/dL)	FLC K (mg/dL)	FLC L (mg/dL)	FLC K/L ratio	BM and PCs %
3/2022	14.0	2.4	134	N/A	0.17	1.17	3.80	0.31	N/A
6/2022	13.7	2.8	167	N/A	0.38	1.50	11.26	0.13	N/A
9/2022	11.9	1.7	122	N/A	0.58	1.17	29.01	0.04	N/A
Initial diagnosis (9/2022)	12.6	2.2	111	8	N/A	N/A	N/A	N/A	32
**9/2022- VEN/AZA start**									
10/2022	8.6	0.4	54	N/A	0.28	0.56	4.68	0.12	N/A
1/2023	8.1	1.0	40	N/A	0.17	0.47	2.75	0.17	15
3/2023	7.9	0.8	33	N/A	0.16	0.91	4.67	0.19	N/A
Post four cycles of VEN/AZA (5/2023)	9.9	1.6	56	< 0.1	0.10	0.10	0.37	0.27	0
9/2023	8.5	2.0	52	N/A	N/A	0.30	0.25	1.20	N/A
11/2023	10.3	0.7	33	N/A	N/A	0.38	0.55	0.69	N/A
Post nine cycles of VEN/AZA (2/2024)	9.7	0.3	28	1.5	N/A	N/A	N/A	N/A	0
3/2024	7.1	0.2	34	N/A	N/A	0.40	0.35	1.14	N/A
Post nine cycles of VEN/AZA (4/2024)	7.1	0.6	21	87	N/A	0.54	0.40	1.35	0

*Note:* N/A represents where values were unavailable. There is a clear decrease in M spike, and bone marrow plasma cell %, following the initiation of VEN/AZA 9/2022. Bone marrow blast% also decreases with VEN/AZA initiation until her disease relapsed.

Abbreviations: (BM blasts %) bone marrow blasts %; (BM PCs %) bone marrow plasma cell %, (Hgb) hemoglobin; free light chain (K) kappa; free light chain (L) lambda; free light chain (K/L), kappa/lambda ratio; (M spike) M protein on serum protein electrophoresis; (Plts) platelets; (WBC) white blood cell.

After 3 years of Lenalidomide maintenance, her M spike began to rise. She was screened for a VEN clinical trial given her known t(11; 14) MM. Screening bone marrow biopsy in 9/2022 revealed relapsed/refractory plasma cell neoplasm with 32% plasma cells in a cellular marrow (Table 1). Surprisingly, it also showed mild myeloid and megakaryocytic atypia, and 8% blasts (Table 1) consistent with MDS with excess blasts 1. Accompanying myeloid NGS panel noted mutations in DNMT3A (variant allele frequency/VAF 10.4%), NRAS (VAF 4.3%), and a variant of uncertain significance (VUS) in DDX41 (VAF 50.9%) (Table 2). DNMT3A and NRAS mutations raised concern for high‐risk MDS. The AML‐FISH panel was normal.

**Table 2:** Changes in blasts percentage and myeloid next generation sequencing (NGS) mutational burden pre‐ and post‐Venetoclax (VEN) and Azacitidine (AZA) treatment.

Evaluation timing	Bone marrow pathology blast (%)	Bone marrow flow cytometry blast (%)	Myeloid NGS panel molecular alterations	Variant allele frequency (%)
Initial diagnosis (9/2022)	8	3	NRAS(NM_002524) c.183A > C(p.Gln61His)	4.3
			DNMT3A(NM_022552)c.2644C > T(p.Arg882Cys)	10.4
			DDX41(NM_016222)c.401A > G(p.Lys134Arg) (VUS)	50.9

*9/28/2022-VEN/AZA start*				
Post two cycles of VEN/AZA (1/2023)	—	—	DDX41(NM_016222)c.401A > G(p.Lys134Arg) (VUS)	50.7

Post four cycles of VEN/AZA (5/2023)	< 0.1	< 0.1	SF3B1(NM_012433)c.1998G > C(p.Lys666Asn)	4.3
			DDX41(NM_016222)c.401A > G(p.Lys134Arg) [ VUS]	50

Post nine cycles of VEN/AZA (2/2024)	1.5	1.5	DNMT3A(NM_022552.5)c.1401_1402insT(p.Lys468Ter)	2.3
			SF3B1(NM_012433.2)c.1998G > C(p.Lys666Asn)	2.5
			BRAF(NM_004333.6)c.1454T > G(p.Leu485Trp)	2.4
			IKZF1(NM_006060.6)c.356G > T(p.Cys119Phe) (VUS)	5.6
			DNMT3A(NM_022552.4)c.2644C > T(p.Arg882Cys)	7.5
			DDX41(NM_016222.2)c.401A > G(p.Lys134Arg) (VUS)	50

Post nine cycles of VEN/AZA (4/2024)	87	82	PTPN11(NM_002834)c.1508G > C(p.Gly503Ala)	2.8
			PTPN11(NM_002834)c.1382C > G(p.Ala461Gly)	3
			IKZF1(NM_006060)c.356G > T(p.Cys119Phe) (VUS)	7.3
			NRAS(NM_002524)c.35G > C(p.Gly12Ala)	21.4
			IKZF1(NM_006060)c.394_395insGCCTGC (p.Val131_Leu132insArgLeu) (VUS)	40.9
			DNMT3A(NM_022552)c.2644C > T(p.Arg882Cys)	45.8
			DDX41(NM_016222)c.401A > G(p.Lys134Arg) (VUS)	50.5

*Note:* There was a decrease in blasts % following VEN/AZA treatment initiation in 9/2022 until her disease relapse. The myeloid NGS panel demonstrates a variant of unknown significance (VUS) in DDX41 with a stable VAF throughout. DNMT3A and NRAS disappear with initial treatment but recur at disease relapse in 2/2024 and 4/2024, respectively. There is an emergence of new mutations demonstrating higher mutational burden with disease relapses with mutations in SF3B1, BRAF, and two VUS in IKZF1.

In 9/2022, she started on treatment for high‐risk MDS and t(11; 14) MM with Azacitidine (AZA) 75 mg/m2 (5 days), VEN 100 mg daily (14 days, with concomitant posaconazole), and weekly Dexamethasone (20 mg). Table 1 shows the decrease in bone marrow blast % and the M spike following treatment initiation. A repeat bone marrow biopsy after four cycles in 5/2023 showed no evidence of disease with 0% plasma cells in the bone marrow and < 0.1% bone marrow blasts (Table 1). The myeloid NGS panel was negative for DNMT3A and NRAS (Table 2). DDX41 VAF was similar to prior, but there was a new SF3B1 mutation (VAF 4.3%) (Table 2). She completed nine cycles of treatment, with additional cycles held due to persistent cytopenias. There was an increased molecular alteration burden on the myeloid NGS panel from the 2/2024 bone marrow biopsy (Table 2).

A repeat bone marrow biopsy, roughly 19 months following the MM relapse and MDS diagnosis (4/2024), was negative for a plasma cell neoplasm, but there were 87% blasts noted (Table 1), along with a greater molecular alteration burden on the myeloid NGS panel (Table 2). On flow cytometry, blasts were noted to be CD19+, CD33+, CD22+, CD34+, TdT+, and MPO + consistent with a mixed phenotype acute leukemia (B/myeloid). Given CD22 positivity, she was transitioned to inotuzumab ozogamicin monotherapy with subsequent complete morphological remission. With no further VEN or MM‐based therapy, her myeloma remained in remission indefinitely.

## 3. Discussion

In summary, patients with MM are at a higher risk of secondary myeloid malignancies which can negatively impact survival in the era of significant treatment advances in MM. Multiple factors influence the development of secondary myeloid malignancies in MM patients including patient‐related factors (e.g., increasing age, male sex, environmental factors), disease‐related factors (e.g., baseline immunophenotypic alterations like those seen in myeloid malignancies in bone marrow stem cells in MM), and treatment‐related factors (e.g., dysplasia secondary to alkylating agents like melphalan and cyclophosphamide, autologous hematopoietic stem cell transplant and immunomodulatory drugs like Lenalidomide) [2, 10–14].

Simultaneous treatment of these hematologic malignancies can be challenging. Regimens such as Lenalidomide/Bortezomib/Dexamethasone, Lenalidomide/Dexamethasone, and Azacitidine/Lenalidomide/Daratumumab have been used. Another approach to treatment is with the BCL‐2 inhibitor VEN, as we have presented in the case above [15, 16]. VEN is routinely used in AML and also has been used in high‐risk MDS and t(11; 14) MM. In AML, the practice‐changing VIALE‐A randomized control trial showed superior survival outcomes with AZA/VEN compared to AZA/placebo in patients who were not candidates for intensive chemotherapy [3]. In high‐risk MDS, the addition of VEN to a hypomethylating agent has been noted to lead to superior survival outcomes compared to treatment with a hypomethylating agent alone, with its efficacy investigated in Phase 3 trials like the VERONA trial (NCT04401748) [4], although a recent update did not find an overall survival benefit with VEN in combination with a hypomethylating agent [17].

The BELLINI and CANOVA trials highlight the use of VEN in t(11;14) MM. In the BELLINI trial, patients with relapsed/refractory t(11; 14) MM were randomly assigned to VEN 800 mg daily or placebo with Bortezomib 1.3 mg/m2 every 21 days for the first 8 cycles and then every 35 days. An 84% overall risk reduction was seen in the VEN group compared to a 70% risk reduction in the placebo group. Greater survival was also seen in the VEN group [5]. In the CANOVA trial, patients were randomized to VEN 800 mg daily or Pomalidomide 4 mg daily with weekly Dexamethasone, and greater clinical efficacy was seen for patients in the VEN group [7]. These two trials illustrate the efficacy of VEN in t(11;14) MM, consistent with our case.

Our work adds to the growing body of literature, as we observed efficacy against myeloma at lower doses of VEN (100 mg with posaconazole, effectively 400mg) than what is described in the BELLINI and CANOVA trials. This could reflect greater sensitivity to VEN in t(11; 14) MM. However, there was a loss of response to VEN‐based therapy after nine cycles with progressive disease, clear from the emergence of 87% blasts on the 4/2024 bone marrow biopsy (Table 1). This was accompanied by clonal evolution with the emergence of mutations in SF3B1, DNMT3A, NRAS, and two separate mutations in IKZF1. Flow cytometry further demonstrated progression to a mixed phenotype acute leukemia (B/myeloid).

We hypothesize that this disease progression could have been driven by the two separate mutations noted as variants of unclear significance in IKZF1 (variant allele frequencies/VAF 7.3% and 40.9%) given the known association between IKZF1 mutations and poor outcomes in B‐cell acute lymphoblastic leukemia [18]. NRAS (VAF 21.4%) portends intermediate‐risk AML according to the European LeukemiaNet 2024 guidelines [19] and is also associated with VEN resistance [20], thus likely was also contributory to progressive disease in this case. SF3B1 mutations are seen in AML with myelodysplasia‐related changes, but with the low VAF (4.3% ⟶ 2.5%) and eventual disappearance on the 4/2024 myeloid NGS, its role in disease progression and loss of response to VEN‐based therapy remains unknown.

In conclusion, MM patients with a concurrent diagnosis of a myeloid malignancy can be managed by treating both disorders simultaneously. Approaching a patient with both diagnoses requires a systematic approach including serial assessment of disease status, prioritization of treatment of the clone with actionable genetic lesions, attention to clone‐specific complications, and a stepwise approach to simultaneously treating both clonal disorders [21]. Regimens against both clonal populations can be considered, and the BCL‐2 inhibitor VEN is one such example.

Our above‐described case report is novel, as VEN appeared to be effective against both malignancies at a lower dose than the effective doses of VEN described in randomized controlled trials of VEN in t(11; 14) MM which is a strength of this case. It remains to be seen if this finding is generalizable to other patients. Our findings are promising, as lower doses of VEN can potentially be offered to patients in similar scenarios, limiting treatment‐related toxicities that can be seen at higher doses that can negatively impact quality of life.

## Ethics Statement

The patient provided consent for the publication of this case report. See signed consent form in supporting documents.

## Disclosure

T.W.L. is a Scholar in Clinical Research of Blood Cancer United.

## Conflicts of Interest

C.G.: BMS‐Advisory Board, Speaker; GSK‐Honorarium; Karyopharm‐Advisory Board, Speaker; Sanofi‐Advisory Board, Speaker; Pfizer‐Advisory Board, Speaker; Janssen‐Advisory Board, Speaker. T.W.L.: Honoraria for consulting/advisory boards from AbbVie, Agilix, Agios/Servier, Apellis, Astellas, BMS/Celgene, Daiichi‐Sankyo, Genentech, Geron, Gilead, GSK, Lilly, Menarini‐Stemline, Novartis, Pfizer, and Syndax; speaking‐related honoraria from AbbVie, Agios/Servier, Astellas, BMS/Celgene, GSK, Incyte, Menarini‐Stemline and Rigel; equity interest in Dosentrx and ThymeCare (stock options in privately held companies); royalties from UpToDate; research funding from AbbVie, AstraZeneca, BMS, Deverra Therapeutics/Coeptis, GSK, Jazz. Pharmaceuticals, Novartis, and Seattle Genetics. The remaining authors declare no conflicts of interest.

## Author Contributions

Conceptualization: Allison O. Taylor and Thomas W. LeBlanc. Data curation: Allison O. Taylor. Writing–original draft preparation: Allison O. Taylor. Writing–review and editing: Allison O. Taylor, Stephanie Clune, JoAnn Liu, Cristina Gasparetto, and Thomas W. LeBlanc. Supervision: Thomas W. LeBlanc.

## Funding

Allison O. Taylor’s work was supported by the American Society of Hematolgy, Hematology Inclusion Pathway Fellow Award, and Conquer Cancer.

## Data Availability

Data used is confidential with consent given only to authors to publish. Data has not been deposited into a publicly available repository.
